# Clinicopathologic and Imaging Features of Non-Small-Cell Lung Cancer with *MET* Exon 14 Skipping Mutations

**DOI:** 10.3390/cancers11122033

**Published:** 2019-12-17

**Authors:** Subba R. Digumarthy, Dexter P. Mendoza, Eric W. Zhang, Jochen K. Lennerz, Rebecca S. Heist

**Affiliations:** 1 Department of Radiology, Massachusetts General Hospital, Boston, MA 02114, USA; dpmendoza@mgh.harvard.edu (D.P.M.); ewzhang@mgh.harvard.edu (E.W.Z.); 2 Center for Integrated Diagnostics, Department of Pathology, Massachusetts General Hospital, Boston, MA 02114, USA; JLennerz@partners.org; 3 Massachusetts General Hospital Cancer Center and Department of Medicine, Massachusetts General Hospital, Boston, MA 02114, USA; RHeist@partners.org

**Keywords:** lung cancer, *MET* exon 14 skipping, mutation, radiology

## Abstract

*MET* exon 14 (*MET*ex14) skipping mutations are an emerging potentially targetable oncogenic driver mutation in non-small-cell lung cancer (NSCLC). The imaging features and patterns of metastasis of NSCLC with primary *MET*ex14 skipping mutations (*MET*ex14-mutated NSCLC) are not well described. Our goal was to determine the clinicopathologic and imaging features that may suggest the presence of *MET*ex14 skipping mutations in NSCLC. This IRB-approved retrospective study included NSCLC patients with primary *MET*ex14 skipping mutations and pre-treatment imaging data between January 2013 and December 2018. The clinicopathologic characteristics were extracted from electronic medical records. The imaging features of the primary tumor and metastases were analyzed by two thoracic radiologists. In total, 84 patients with *MET*ex14-mutated NSCLC (mean age = 71.4 ± 10 years; F = 52, 61.9%, M = 32, 38.1%; smokers = 47, 56.0%, nonsmokers = 37, 44.0%) were included in the study. Most tumors were adenocarcinoma (72; 85.7%) and presented as masses (53/84; 63.1%) that were peripheral in location (62/84; 73.8%). More than one in five cancers were multifocal (19/84; 22.6%). Most patients with metastatic disease had only extrathoracic metastases (23/34; 67.6%). Fewer patients had both extrathoracic and intrathoracic metastases (10/34; 29.4%), and one patient had only intrathoracic metastases (1/34, 2.9%). The most common metastatic sites were the bones (14/34; 41.2%), the brain (7/34; 20.6%), and the adrenal glands (7/34; 20.6%). Four of the 34 patients (11.8%) had metastases only at a single site. *MET*ex14-mutated NSCLC has distinct clinicopathologic and radiologic features.

## 1. Introduction

Targeted therapy using small-molecule tyrosine kinase inhibitors (TKIs) has become the standard of treatment in non-small-cell lung cancer (NSCLC) harboring specific driver alterations in the epidermal growth factor (*EGFR*), anaplastic lymphoma kinase (*ALK*), rearrangement of the receptor tyrosine kinase 1 (*ROS1*), and V-raf murine sarcoma viral oncogene homolog B (*BRAF*) genes [1,2], demonstrating improved survival and quality-of-life benefits [3,4,5,6,7,8,9,10,11,12,13]. However, the driver alterations of a majority of lung adenocarcinomas are unknown [14,15,16,17], highlighting the importance of identifying new targetable mutations. Mesenchymal–epithelial transition (*MET*) gene exon 14 skipping has emerged as a promising oncogenic target in lung cancer.

The *MET* proto-oncogene is located on chromosome 7q21-q31 and encodes a receptor tyrosine kinase implicated in RAS/MAPK, Rac/Rho, and PI3K/Akt signaling pathways, mediating cellular growth, development, anti-apoptosis, and metastasis [18,19,20]. First described in the 1980s [21,22], *MET* amplification and overexpression have been found in a wide variety of malignancies, including colon, breast, liver, gastric cancers, and sarcomas [23,24]. Overexpression of *MET* has also been implicated in 25–70% of all NSCLC [14] and as an acquired resistance mechanism in *EGFR*-mutated NSCLC [25].

*MET* exon 14 (*MET*ex14) skipping is found in approximately 3–4% of NSCLC [14,26] and represents a unique subset of all *MET* mutations, whereby DNA mutations affect RNA splicing sites and result in the loss of the CBL-E3 ligase binding site and sustained activation of the *MET* receptor [27]. Importantly, *MET*ex14 skipping and other NSCLC driver alterations, including *EGFR*, *ALK*, and *ROS1* mutations, are mutually exclusive [26,28]. Furthermore, *MET*ex14 skipping has also demonstrated responsiveness to targeted *MET* therapies, making it currently the most predictive biomarker of the sensitivity to *MET* TKIs [29,30,31]. Small-molecule TKIs, such as crizotinib and cabozantinib, have shown promise in the treatment of NSCLC harboring *MET*ex14 mutations, in various case reports [29,30,32]. In addition, multiple clinical trials currently underway, investigating novel *MET* TKIs such as tepotinib and capmatinib, have demonstrated promising preliminary results [33,34,35,36,37,38].

Several studies have investigated the imaging features that may predict the presence of *EGFR*, *ALK*, *ROS1*, and other potentially targetable mutations in NSCLC [39,40,41,42,43,44,45,46,47,48]. To our knowledge, however, no study has systematically assessed the radiologic features of NSCLC harboring primary *MET*ex14 skipping (*MET*ex14-mutated NSCLC). The goal of this study was to determine the clinicopathologic and radiologic features that may suggest the presence of *MET*ex14 skipping in NSCLC.

## 2. Results

### 2.1. Clinicopathologic Characteristics

The clinicopathologic characteristics for patients with *MET*ex14-mutated NSCLC are summarized in Table 1. Most patients were female (52/84; 61.9%), and more than half were either previous or current smokers (47/84; 56%). In our cohort, there was equal distribution of stage 1 (34/84; 40.5%) and stage 4 (34/84; 40.5%) disease. A vast majority of the tumors were adenocarcinoma (72/84; 85.7%), followed by squamous (6/84; 7.1%) and sarcomatoid (3/84; 3.6%) carcinomas.

### 2.2. Imaging Features of the Primary Tumor

The imaging features of the primary tumor in *MET*ex14-mutated NSCLC are summarized in Table 2. Most tumors presented as masses measuring 3 cm or more (53/84; 63.1%) and were predominantly located in the upper lobes (59/84; 70.2%) at the periphery (62/84; 73.8%). Approximately one-third of the tumors were either part-solid (21/84; 25.0%) or pure ground-glass (6/84; 7.1%), while the rest were solid (57/84; 67.9%). Approximately one in five of the patients had multifocal tumors (19/84; 22.6%).

### 2.3. Metastatic Patterns

In patients with metastatic *MET*ex14-mutated NSCLC, there was a higher propensity for extrathoracic metastases (28/34; 82.4%) compared to intrathoracic metastases (13/34; 38.2%). Most patients only had extrathoracic metastases (23/34; 67.6%), while only one (2.9%) had only intrathoracic metastases, and the rest had both intrathoracic and extrathoracic metastases (10/34; 29.4%). The most common metastatic sites were the bones (14/34; 41.2%), the brain (7/34; 20.6%), and the adrenal glands (7/34; 20.6%). Four of the 34 patients (11.8%) with metastatic disease had a single site of metastasis. Patterns of lymphadenopathy and metastases are presented on Table 3.

## 3. Discussion

We present the first systematic assessment of the imaging features and patterns of metastasis in NSCLC with *MET*ex14 skipping mutations. We found that *MET*ex14-mutated NSCLC tumors commonly present as peripheral masses and that a considerable proportion of patients had multifocal lung cancer at presentation. In addition, among patients with metastatic disease, extrathoracic metastases were common, with the most common sites being the bones, brain, and adrenal glands.

**Figure 1:**
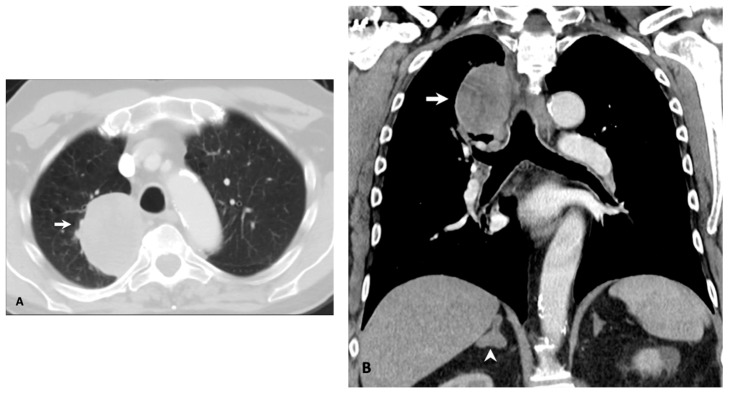
Oligometastatic NSCLC with sarcomatoid histology in a 70-year-old male former smoker. (**A**) Axial computed tomography (CT) image shows a large solid mass in the right upper lobe (**A**, arrow). (**B**) Coronal CT image shows the right upper lobe mass (**B**, arrow) and a right adrenal nodule (**B**, arrowhead). Biopsies and molecular testing of the right upper lobe mass and adrenal metastasis confirmed the presence of a *MET*ex14 skipping mutation.

In our cohort, the average age of 71.4 years was higher than those previously reported for other targetable driver mutations [46,48,49]. This advanced age was reported in another study, which found a similar median age of 72.5 years [26]. In our cohort, the mutation did not appear to have a gender predilection and affected smokers and nonsmokers nearly evenly, in contrast to *EGFR* and *ALK* mutations that are more common in never and light smokers. From a pathological standpoint, a vast majority of the tumors were adenocarcinoma. Although still rare, there was a relatively increased frequency of sarcomatoid carcinoma (3.6%; Figure 1). While *MET*ex14 alterations have been described in up to 4% of lung cancers, they can be seen in approximately one-third of sarcomatoid carcinoma, which has generally been associated with a poorer prognosis [50].

With respect to imaging features, in our cohort, most of the primary *MET*ex14-mutated tumors were solid masses that were typically located in the periphery of the upper lobes. Air bronchograms, cavitation, and cystic changes were seen in less than 5% of the tumors. These features are not unique to *MET*ex14-mutated NSCLC and have been described in other mutated NSCLC, including those with *ALK* or *ROS1* rearrangements [42,45,48,51]. This, however, is in contrast to *EGFR*-mutated NSCLC, reported to have increased incidence of subsolid and pure ground-glass lesions and increased frequency air bronchogram in the tumor [41]. For instance, our group has previously reported the presence of air bronchograms in up to 28% of *EGFR*-mutated NSCLC tumors [46], in contrast to their presence in less than 4% of *MET*ex14-mutated tumors in our current cohort.

**Figure 2:**
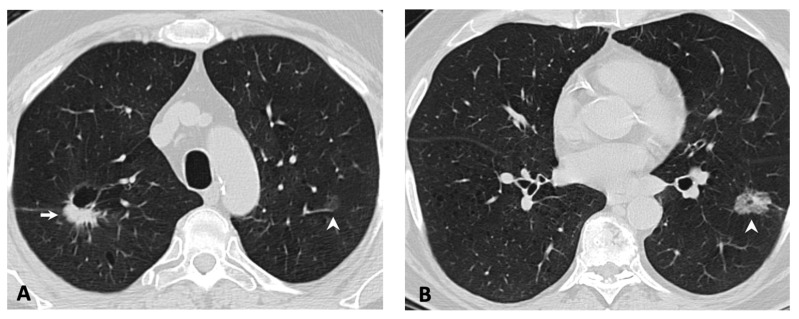
Multifocal lung adenocarcinomas in an 80-year-old female former smoker. (**A**) Axial CT through the upper lobes shows a part-cystic, part-solid nodule in the right upper lobe (**A**, arrow) and a faint ground-glass nodule in the left upper lobe (**A**, arrowhead). (**B**) Axial CT slice through the lower lobes shows an additional ground-glass nodule in the left lower lobe (**B**, arrowhead). Findings are consistent with multifocal adenocarcinomas. The patient went on to have a right upper lobectomy. Pathology and molecular testing revealed an adenocarcinoma with *MET*ex14 skipping mutation.

While there was no primary tumor imaging feature unique to *MET*ex14-mutated NSCLC in our cohort, there was an increased frequency of multifocality, with more than one in five patients having multifocal adenocarcinoma at the time of initial presentation (Figure 2). This incidence was higher than the prevalence of *ALK* and concomitant *ALK* and *EGFR* alterations in multifocal lung adenocarcinomas [52]. It is possible that the multifocality reflects multiple synchronous adenocarcinomas with distinct splice site mutations, a finding which has been previously described in the literature for *MET*ex14-mutated primary lung adenocarcinomas [53]. Multifocal adenocarcinomas at presentation may help indicate the possibility of *MET*ex14-mutated NSCLC and lead to more rapid triaging for molecular screening. These synchronous lung adenocarcinomas are increasingly being recognized and can be a diagnostic and management challenge [52]. Detection of these potentially targetable mutations in multifocal NSCLC may prove to be beneficial when these malignancies progress and metastasize.

With respect to metastatic patterns, in our cohort, there were twice as many patients who had extrathoracic metastases compared to those with intrathoracic metastases. Most patients also had only extrathoracic metastases without intrathoracic metastases, while only one patient had only intrathoracic metastases without extrathoracic metastases. The most common sites were the bones, brain, and adrenal glands. This pattern of metastasis is in contrast to that of *EGFR*-mutated and *ALK*-rearranged NSCLC, which have been associated with an increased propensity for intrathoracic metastases. For example, in our group’s previous work, we reported a frequency of 69% for lung metastases in patients with *EGFR*-mutated NSCLC, which tended to be diffuse and miliary-like when present [46]. In comparison, the frequency of lung metastases in our cohort of patients with *MET*ex14-mutated NSCLC was less than 15%. Similarly, we reported frequencies of lymphangitic carcinomatosis of 37% in patients with *ALK*-rearranged NSCLC [45] and of 42% in those with *ROS1*-rearranged NSCLC [48], in comparison to less than 12% in our current cohort. 

The high propensity for brain metastases is a feature that has also been reported for tumors with other potentially targetable mutations, including *EGFR*, *ALK*, *ROS1*, and *RET* [54,55,56,57]. An increased frequency of brain metastases has also been reported for *MET*ex14-mutated NSCLC [58]. In our cohort, 20% of patients with metastatic disease had brain metastases at the time of initial diagnosis. In our group’s previous works, we reported frequencies of brain metastases of 40% in patients with *EGFR* mutations [46], 24% in those with *ALK* rearrangements [45], 10% in those with *BRAF* mutations [44], and 9% in those with *ROS1* rearrangements [48]. The high incidence of brain metastases in these mutational subgroups highlights the need for agents that can reliably penetrate the blood–brain barrier.

Notably, there were four patients (11.8%) who only had one site of metastasis (i.e., oligometastatic disease). Three of the patients had only adrenal metastases (Figure 1), while one patient only had a soft tissue metastasis. Although a standardized, unified definition for oligometastatic NSCLC has yet to be agreed on, several studies have reported improved outcomes in this subset of patients with limited metastatic burden when subjected to radical treatment with curative intent [59,60]. The true incidence of oligometastatic NSCLC is unknown, which is largely due to the lack of a precise definition. The relatively high incidence of oligometastatic disease in our cohort may partly be a result of referral bias, although further study as to the possible association with *MET*ex14 skipping mutations should be considered. To date, no specific molecular genotype has been associated with oligometastatic NSCLC.

Our study has several limitations. Although this is the largest study to date to assess the imaging features and metastatic patterns in *MET*ex14-mutated NSCLC, our cohort was still relatively small due to the rarity of *MET*ex14 skipping mutations in NSCLC overall. The data were collected retrospectively from a single institution, predisposing to selection and referral bias and potentially limiting its generalizability to larger populations. Despite these limitations, our findings add to the growing understanding of the clinical and radiologic features of *MET*ex14-mutated NSCLC.

## 4. Materials and Methods

### 4.1. Patient Identification and Selection

Under an institutional review board-approved protocol (Partners Human Research protocol number 2019P000198), we identified patients who presented to our thoracic medical oncology clinic between January 2013 and December 2018, who met the following criteria: (1) confirmed non-small-cell lung cancer by histology; (2) confirmed *MET*ex14 skipping found in the primary tumor or a metastatic lesion; and (3) availability of pre-treatment imaging data for review, obtained either at our institution or at another institution, with the images uploaded into our picture-archiving and communication system (AGFA Impax 6, Mortsel, Belgium). We collected clinicopathologic data, including age, sex, smoking history, tumor histology, and stage of disease at the time of diagnosis.

### 4.2. Molecular Testing

Molecular testing was performed on tissue samples obtained from either the primary lung tumor or a metastatic lesion. *MET*ex14-skipping status was determined using anchored multiplex PCR (AMP) based on next-generation sequencing.

The laboratory-developed test was performed in a Clinical Laboratory Improvement Amendments (CLIA)-certified laboratory, and its performance was validated for samples showing tumor purity of 5% or higher.

### 4.3. Imaging Protocol and Image Analysis

All patients had imaging studies performed prior to the initiation of cancer-specific treatment. All computed tomography (CT) examinations were performed on multidetector CTs utilizing helical acquisition with automatic exposure control or fixed mA and tube potentials of up to 120 kV. Unless contraindicated, iodinated intravenous contrast was routinely administered. Positon emission tomography (PET) images when available (*n* = 53/84) was also reviewed.

CT images closest to diagnosis and prior to any anti-cancer treatment were selected for review. An experienced thoracic radiologist and a fellow in thoracic imaging (SRD and DM) reviewed the images concurrently, and imaging findings were determined and recorded by consensus.

CT features of the primary lung tumor, when identifiable, and patterns of metastases were assessed. The features of the primary tumor that were assessed were: size, density (solid, mixed, ground-glass), location (lobar location and central versus peripheral), and the presence of cavitation, cystic changes, air bronchograms, or calcifications. The tumors involving or at the lobar bronchus were considered central tumors.

The lymph nodes that measured greater than 10 mm in the short axis and/or with increased fluorodeoxyglucose uptake in PET imaging were considered malignant. The presence of metastases in the lungs, pleura, bones, brain, liver, adrenal glands, and other visceral organs was also documented following a review of other imaging studies, including a CT of the abdomen and pelvis, a CT or magnetic resonance imaging (MRI) of the brain, and a whole-body PET. Note was also made of pulmonary lymphangitic carcinomatosis. When available, imaging findings were also correlated with surgical pathology to verify nodal and distant metastases.

## 5. Conclusions

NSCLC with primary *MET*ex14 skipping mutations more commonly affected older individuals, without preponderance with respect to sex or smoking status. The primary tumors in NSCLC with primary *MET*ex14 skipping mutations tended to present as solid, peripheral masses. While the tumors harboring these mutations mostly have adenocarcinoma histology, there was an increased frequency of tumors with sarcomatoid features. There was also a high frequency of multifocality and extrathoracic metastases, commonly affecting bones, brain, and adrenal glands. A combination of these clinicopathologic and imaging features may suggest the presence of *MET*ex14-mutated NSCLC and help identify the subset of patients who may benefit from further molecular genotyping.

## Figures and Tables

**Table 1:** Clinicopathologic characteristics of patients with *MET*ex14- NSCLC (*n* = 84).

Clinical Characteristics		
**Age**		
Mean, SD (in years)	71.4	10
Median, range (in years)	72.5	43–89
**Sex**	***n***	**%**
F (%)	52	61.9
M (%)	32	38.1
**Smoking status**		
Never	37	44.0
Current/Previous	47	56.0
**Stage**		
I	34	40.5
II	9	10.7
IIIA	5	6.0
IIIB	2	2.4
IV	34	40.5
**Histology**		
Adenocarcinoma	72	85.7
Squamous	6	7.1
Sarcomatoid carcinoma	3	3.6
Others	3	3.6

**Table 2:** Imaging features of the primary tumor in *MET*ex14-mutated NSCLC (*n* = 84).

Tumor Imaging Features		
**Size**		
Mean, SD (in mm)	40.8	21.4
Median, range (in mm)	34.5	10–109
**Size**	***n***	**%**
Mass (>3 cm)	53	63.1
Nodule (≤3 cm)	31	36.9
**Lobar location**		
RUL	38	45.2
RML	4	4.8
RLL	10	11.9
LUL	17	20.2
LLL	15	17.9
**Lobar location**		
Upper/Middle	59	70.2
Lower	25	29.8
**Axial location**		
Peripheral	62	73.8
Central	22	26.2
**Density**		
Solid	57	67.9
Part-solid	21	25.0
Pure ground-glass	6	7.1
**Margin**		
Smooth	9	10.7
Lobulated	53	63.1
Spiculated	22	26.2
**Other Tumor features**		
Air bronchograms	3	3.6
Cavitation	4	4.8
Cystic component	4	4.8
Calcification	0	0.0
**Multifocal**	19	22.6

RUL: right upper lobe; RML: right middle lobe; RLL: right lower lobe; LUL: left upper lobe; LLL: left lower lobe.

**Table 3:** Patterns of lymphadenopathy and metastases in patients with stage IV *MET*ex14-mutated NSCLC (*n* = 34)。

Metastatic Site	*n*	%
**Nodal metastasis**		
Ipsilateral hilar	29	85.3
Ipsilateral mediastinal	19	55.9
Contralateral hilar/mediastinal	12	35.3
Supraclavicular	7	20.6
**Intrathoracic metastasis**	13	38.2
Lung	5	14.7
Lymphangitic carcinomatosis	4	11.8
Pleural	10	29.4
Pericardial	2	5.9
**Extrathoracic Metastasis**	28	82.4
Adrenal	7	20.6
Liver	3	8.8
Gastric	2	5.9
Splenic	0	0.0
Bone (Lytic)	14	41.2
Brain	7	20.6
Soft tissue	1	2.9
Distant lymph node	4	11.8
**Metastatic Distribution**		
Intrathoracic only	1	2.9
Extrathoracic only	23	67.6
Intra- and extrathoracic	10	29.4
**Number of sites**		
One	4	11.8
Two or more	30	88.2
